# Supplementary material for: Correlates of variability in endurance shuttle walk test time in patients with chronic obstructive pulmonary disease
Source: PLoS One. 2021 Apr 21;16(4):e0249786. doi: 10.1371/journal.pone.0249786 (PMC8059801; doi:10.1371/journal.pone.0249786)
Supplement: S1 Table — (PDF) [file pone.0249786.s001.pdf]

S1 Table. ESWT parameters of the whole group and the three subgroups based on tolerated duration during the ESWT.

Variables	All patients with COPD (n=245)	Group 1 (n=41) Tlim <3 min	Group 2 (n=124) Tlim = 3-8 min	Group 3 (n=80) Tlim >8 min	p-value
Speed (km/h)	4.1 (3.6-4.6)	3.6 (3.1-4.4)	4.1 (3.6-4.6)	4.1 (3.7-5.1)	0.007 [#]
Time (s)	360 (221-617)	147 (118-165)	291 (239-383)	1200 (623-1200)	<0.001 [*,#,†]
SpO_2 rest (%) [a]	96 (94-97)	95 (94-97)	96 (94-97)	96 (94-97)	0.214
SpO_2 at max (%) [b]	90 (85-94)	87 (84-91)	89 (84-94)	92 (89-95)	<0.001 [#,†]
SpO_2 delta (max-rest, %) [c]	-5 (-10- -2)	-8 (-12- -5)	-5 (-11- -2)	-3 (-8- -1)	0.012 [#]
HR rest (bpm) [d]	83 (73-91)	86 (78-92)	82 (73-90)	83 (72-92	0.351
HR at max (bpm) [e]	113 (100-122)	108 (100-120)	115 (100-122)	113 (100-125)	0.574
$HRmax_{ESWT}/HRmax_{ISWT}$ (%) [e]	99 (93-106)	97 (89-103)	98 (92-104)	103 (96-111)	0.027
HR delta (max-rest, bpm) [f]	29 (20-39)	26 (18-33)	29 (19-40)	31 (23-40)	0.188
Borg score dyspnoea rest [g]	2 (1-3)	2 (1-3)	2 (1-3)	1 (1-2)	<0.001 [#,†]
Borg score dyspnoea max [h]	5 (4-7)	6 (5-8)	5 (4-7)	4 (3-6)	<0.001 [#,†]
Borg score dyspnoea delta [g]	3 (2-5)	4 (3-5)	4 (2-5)	3 (2-5)	0.237
Borg score leg fatigue rest [g]	2 (1-3)	2 (1-3)	2 (1-3)	2 (1-3)	0.227
Borg score leg fatigue max [h]	5 (3-6)	5 (4-7)	5 (3-6)	4 (3-5)	0.181
Borg score leg fatigue delta [g]	3 (1-4)	3 (2-4)	2 (1-4)	3 (1-4)	0.292

*Data is presented as median (IQR 25-75%). * indicates a significant difference after Bonferroni post-hoc correction between group 1 and group 2, [#] indicates a significant difference after Bonferroni post-hoc correction between group 1 and group 3, [†] indicates a significant difference after Bonferroni post-hoc correction between group 2 and group 3. Alphabetic characters in superscript indicate a sample size deviant from n = 245 (group 1: 41, group 2: 124, group 3: 80) with the following: a. n = 236 (41, 123, 72), b. n = 236 (41, 122, 73), c. n = 235 (41, 122, 72), d. n = 182 (27, 90, 65), e. n = 181 (27, 88, 66), f. n = 180 (27, 88, 65), g. n = 237 (41, 123, 73), h. n = 238 (41, 124, 73). Definitions of abbreviations: HR = heartrate, $HRmax_{ESWT}/HRmax_{ISWT}$ = maximal HR of the endurance shuttle walk test relative to the maximal HR during the incremental shuttle walk test, SpO_2 = peripheral capillary oxygen saturation, Tlim = tolerated duration.*
