# Supplementary material for: Correlates of variability in endurance shuttle walk test time in patients with chronic obstructive pulmonary disease
Source: PLoS One. 2021 Apr 21;16(4):e0249786. doi: 10.1371/journal.pone.0249786 (PMC8059801; doi:10.1371/journal.pone.0249786)
Supplement: S2 Table — (PDF) [file pone.0249786.s002.pdf]

S2 Table. Univariate linear regression models for the subject characteristics, severity of complaints, pulmonary function and physical performance with the tolerated duration on the ESWT.

Variables	R^2	Df	Beta	CI	P-value
Gender (male (%))	0.003	243	37.847	-55.732- 131.426	0.426
Age (years)	0.030	243	-8.296	-14.235- -2.358	0.006
BMI (kg/m^2)	0.021	243	-9.499	-17.639- -1.359	0.022
CCI	0.001	225	7.599	-32.123- 47.321	0.707
FFMI	0.019	207	-20.585	-40.896- -0.275	0.047
**Severity of complaints**					
mMRC score	0.054	220	-73.405	-114.311- -32.499	<0.001
CAT score	0.000	212	-0.250	-7.503- 7.004	0.946
HADS anxiety score	0.000	219	-0.597	-12.280- 11.086	0.920
HADS depression score	0.001	219	2.357	-10.608- 15.322	0.720
CIS fatigue score	0.000	221	0.246	-6.511- 7.003	0.943
**Pulmonary parameters**					
FEV_1 (L)	0.019	238	94.852	8.328- 181.376	0.032
FEV_1 (% predicted)	0.020	238	3.361	0.377- 6.345	0.027
Tiffeneau index (%)	0.020	238	4.487	0.510- 8.464	0.027
FRC (% predicted)	0.007	233	-0.830	-2.067- 0.408	0.188
RV (% predicted)	0.001	234	-0.264	-1.152- 0.623	0.558
TLC (% predicted)	0.002	234	-0.831	-3.385- 1.723	0.522
FRC/TLC (%)	0.018	233	-5.319	-10.373- -0.265	0.039
RV/TLC (%)	0.011	234	-3.826	-8.489- 0.837	0.107
DL_{CO} (mL/mmHg/min)	0.009	226	22.554	-7.964- 53.071	0.147
DL_{CO} (% predicted)	0.011	226	2.404	-0.624- 5.432	0.119
**Physical parameters**					
Wmax (Watt)	0.054	201	2.534	1.066- 4.003	0.001
VO_2Max (ml/min/kg)	0.120	192	32.856	20.162- 45.551	<0.001
VO_2Max (% predicted)	0.076	192	6.765	3.411- 10.118	<0.001
CWRT time (s)	0.034	171	0.238	0.046- 0.430	0.015
MVC (Nm)	0.004	224	-0.645	-1.914- 0.624	0.317
MVC (% predicted)	0.001	224	-0.803	-4.170- 2.564	0.639
Physical activity (steps/day)	0.059	240	0.037	0.018- 0.056	<0.001
Physical activity (average PAL)	0.060	239	813.292	401.348- 1225.235	<0.001

Definitions of abbreviations: BMI = body mass index, CAT = COPD assessment test, CCI = Charlson Comorbidity Index, CI = confidence interval, CIS = checklist individual strength, CWRT = constant work rate cycle test, Df = degrees of freedom, DL_{CO} = single-breath carbon monoxide diffusion capacity, FEV_1 = forced expiratory volume in 1 second, FFMI = fat free mass index, FRC = functional residual capacity, HADS = hospital anxiety and depression scale, mMRC = modified medical research council, MVC = maximal voluntary contraction, PAL = physical activity level, TLC = total lung capacity, RV = residual volume, VO_2max = maximal oxygen uptake, Wmax = maximal workload.
