# Supplementary material for: Correlates of variability in endurance shuttle walk test time in patients with chronic obstructive pulmonary disease
Source: PLoS One. 2021 Apr 21;16(4):e0249786. doi: 10.1371/journal.pone.0249786 (PMC8059801; doi:10.1371/journal.pone.0249786)
Supplement: S3 Table — (PDF) [file pone.0249786.s003.pdf]

S3 Table. Univariate linear regression models for the ISWT parameters with the tolerated duration on the ESWT.

Variables	R^2	Df	Beta	CI	P-value
Distance (m)	0.029	240	0.449	0.121-0.778	0.008
Distance (% predicted)	0.033	241	1.814	0.561- 3.067	0.005
Speed (km/h)	0.022	243	53.959	8.110-99.808	0.021
SpO_2 rest (%)	0.009	243	15.188	-5.355- 35.731	0.147
SpO_2 max (%)	0.022	243	8.207	1.253-15.161	0.021
SpO_2 delta (max-rest, %)	0.017	243	8.396	0.440-16.353	0.039
HR rest (bpm)	0.003	182	-1.781	-6.555-2.992	0.463
HR max (bpm)	0.009	182	-1.910	-4.857-1.038	0.203
$HRmax_{ISWT}/HRmax_{CPET}$	0.066	165	-6.862	-10.835- -2.889	0.001
HR delta (max-rest, bpm)	0.005	182	-1.821	-5.420-1.777	0.319
Borg score dyspnoea rest	0.021	243	-37.866	-70.270- -5.462	0.022
Borg score dyspnoea max	0.051	243	-40.123	-62.053- -18.192	<0.001
Borg score dyspnoea delta	0.014	243	-20.618	-42.132- 0.897	0.060
Borg score fatigue rest	0.002	243	8.860	-16.147-33.867	0.486
Borg score leg fatigue max	0.019	243	-23.227	-44.285- -2.168	0.031
Borg score leg fatigue delta	0.029	243	-27.962	-48.317- -7.607	0.007

Definitions of abbreviations: CI = confidence interval, Df = degrees of freedom, HR = heartrate, $HRmax_{ISWT}/HRmax_{CPET}$ = maximal HR of the incremental shuttle walk test relative to the maximal HR during the cardiopulmonary exercise test, SpO_2 = peripheral capillary oxygen saturation.
