# Supplementary material for: Correlates of variability in endurance shuttle walk test time in patients with chronic obstructive pulmonary disease
Source: PLoS One. 2021 Apr 21;16(4):e0249786. doi: 10.1371/journal.pone.0249786 (PMC8059801; doi:10.1371/journal.pone.0249786)
Supplement: S4 Table — (PDF) [file pone.0249786.s004.pdf]

S4 Table. Multivariate linear regression analysis to predict tolerated duration on the ESWT.

Variables	Unstandardized B	95% CI for B	P-value
(Constant)	160.235	-601.759-922.226	0.678
BMI (kg/m^2)	-12.312	-23.225- -1.339	0.027
VO_2max (ml/min/kg)	34.907	16.077-53.737	<0.001
CWRT time (s)	0.219	0.018-0.420	0.033
Physical activity (average PAL)	576.920	38.403-1115.436	0.036
ISWT speed	-105.806	-182.810- -28.802	0.007
ISWT Borg score rest dyspnoe	-65.736	-113.438- -18.033	0.007
ISWT Borg score delta leg fatigue	-32.033	-57.387- -6.680	0.014

Definitions of abbreviations: B = beta, BMI = body mass index, CI = confidence interval, CWRT = constant work rate cycle test, ISWT = incremental shuttle walk test, PAL = physical activity level, VO_2max = maximal oxygen uptake.
